# The Impact of Infant Feeding Regimen on Cow’s Milk Protein Allergy, Atopic Dermatitis and Growth in High-Risk Infants during the First 6 Months of Life: The Allergy Reduction Trial

**DOI:** 10.3390/nu15112622

**Published:** 2023-06-03

**Authors:** Theodora Boutsikou, Mikaela Sekkidou, Eva Karaglani, Adamantia Krepi, George Moschonis, Nicolaos Nicolaou, Nicoletta Iacovidou, Rouzha Pancheva, Miglena Marinova-Achkar, Simoneta Popova, Anastasia Kapetanaki, Zoi Iliodromiti, Vassiliki Papaevangelou, Olympia Sardeli, Evangelia Papathoma, Anne Schaafsma, Rolf Bos, Yannis Manios, Paraskevi Xepapadaki

**Affiliations:** 1 Neonatal Department, Medical School, National and Kapodistrian University of Athens, Aretaieio Hospital, 11528 Athens, Greece; mandokr1@gmail.com (A.K.); niciac58@gmail.com (N.I.); ziliodromiti@yahoo.gr (Z.I.); 2 Asthma and Allergy Center, 3025 Limassol, Cyprus; miksekk@gmail.com (M.S.); nic.nicolaou@googlemail.com (N.N.); 3 University of Nicosia Medical School, 2408 Nicosia, Cyprus; 4 Department of Nutrition and Dietetics, School of Health Science and Education, Harokopio University, 70 El. Venizelou Ave., 17671 Athens, Greece; evkaraglani@gmail.com (E.K.); yannis.manios@gmail.com (Y.M.); 5 Department of Food, Nutrition and Dietetics, School of Allied Health, Human Services and Sport, La Trobe University, Melbourne, VIC 3086, Australia; g.moschonis@latrobe.edu.au; 6 Department of Hygiene and Epidemiology, Faculty of Public Health, Medical University of Varna, 9000 Varna, Bulgaria; rouzha.pancheva@gmail.com (R.P.); mig_mar@abv.bg (M.M.-A.); simonetapopova@gmail.com (S.P.); 7 Neonatal Intensive Care Unit, General and Maternity Hospital Elena Venizelou, 11521 Athens, Greece; pednancy@hotmail.com; 8 Third Department of Pediatrics, National and Kapodistrian University of Athens, ATTIKON General University Hospital, 12462 Athens, Greece; vpapaev@gmail.com (V.P.); ol.sardeli@googlemail.com.com (O.S.); 9 Neonatal Intensive Care Unit, Alexandra General Hospital, 11528 Athens, Greece; lilpapaki@yahoo.gr; 10 FrieslandCampina, 3818 LE Amersfoort, The Netherlands; anneschaafsma@gmail.com (A.S.); rolf.bos@frieslandcampina.com (R.B.); 11 Institute of Agri-Food and Life Sciences, Hellenic Mediterranean University Research Centre, 71410 Heraklion, Greece; 12 Allergy Department, 2nd Pediatric Clinic, National and Kapodistrian University of Athens, 15771 Athens, Greece; vickyxepapadaki@gmail.com

**Keywords:** cow’s milk protein allergy, atopic dermatitis, hydrolyzed formula, breastfed infants

## Abstract

The development of early-onset cow’s milk protein allergy and atopic dermatitis during the first months of life is multifactorial, including both genetic and nutritional aspects. This study aims to assess the impact of different feeding patterns on the incidence of cow’s milk protein allergy, atopic dermatitis, and growth among infants with a family history of allergy. A total of 551 high-risk infants were randomly recruited from 3 European countries in three feeding regimens: exclusive breastfeeding, partially hydrolyzed formula, or standard formula with intact protein either exclusively or supplementary to breastfeeding. During the first 6 months of intervention, amongst infants with a family history of atopic dermatitis, 6.5% of partially hydrolyzed formula-fed infants and 22.7% of exclusively breastfed infants (*p* = 0.007) presented with atopic dermatitis respectively. Growth as assessed by weight increase did not differ between the aforementioned groups. Although cow’s milk protein allergy was not related to the different milk feeding regimens in the whole cohort, when adjusting for high breast milk intake, the respective incident was significantly lower in the infants consuming partially hydrolyzed formula (*p* < 0.001). This data indicates that a specific partially hydrolyzed formula could serve as a more appropriate complement to breast milk compared to a standard intact protein formula in high-risk infants, to reduce the incidence of atopic dermatitis.

## 1. Introduction

The prevalence of Cow’s Milk Protein Allergy (CMPA) has increased in developed countries in the last four decades [1]. Current epidemiological data suggest that CMPA affects 1.4–3.8% of infants, according to parental reports [2], while the respective prevalence as assessed by food challenges ranges from 0% to 3% [3]. In parallel, the onset of atopic dermatitis (AD) occurs early in life, with a prevalence of approximately 20% and an incidence of 9.6% among infants and children in Westernized countries [4]. The presence of a family history of atopy and AD have been well recognized as risk factors for CMPA, while CMPA coexists with a substantial proportion of infants with early and severe AD [5].

The diagnostic approach for CMPA includes, depending on the type of reaction, a detailed medical history, allergy work-up, including skin prick tests (SPT) and/or determination of specific IgE(s) to milk proteins, while oral food challenge (OFC) remains the gold standard for CMPA diagnosis. CoMiSS has lately been used as an additional awareness tool in the assessment of subjects with CMPA-suggestive symptoms [6]. With respect to AD, the nine-region Scoring of Atopic Dermatitis Index (SCORAD) is the major tool for the assessment of the disease’s severity [7].

Given the prevalence, comorbidities, and cost of allergic diseases for the healthcare system, primary prevention strategies have been developed with respect to feeding practices for infants at high risk [8]. Exclusive breastfeeding (EBF) is recommended for at least 4 months of age, although the potential protective effect of EBF on the occurrence of allergic disease remains controversial [9,10,11,12]. Recent reports are favorable of a protective effect of EBF on respiratory allergic diseases, during the first years of life. However, strategies for the primary prevention of food allergy (FA) and CPMA are still inconclusive. The introduction of partially hydrolyzed formulas (pHF) in high-risk infants aiming at reducing the development of allergy-associated diseases has been recently challenged [13,14,15]. Noteworthy, early (after the 1–4 postnatal week) and daily supplementation of small amounts of standard cow’s milk formula (SF) with intact protein or hydrolyzed formula to breastfed infants (mixed feeding), has been associated with a reduced risk of milk sensitization and CMPA [16,17]. We have recently reported that a specific partially hydrolyzed whey-based formula (pHF) reduced the risk of AD development, particularly in infants with a family history of AD, and tended to reduce the development of CMPA in non-exclusively breastfed infants at high risk for allergy, as compared to SF [18].

According to the U.S. Food and Drug Administration and European Food Safety Authority (EFSA) requirements, growth monitoring studies (GMS) is necessary to show the safety of formulas with new protein fractions, during the period when formula is the sole source of nutrition. However, the different results in growth velocity among the formula feeding patterns could potentially reflect crucial differences in protein concentrations when weight, length, or head circumference are assessed. With regards to effects on growth in mixed-fed (either pHF or SF with breastmilk) infants, no differences (noninferiority) have been reported, while mixed feeding with pHF closely tracked EBF [19]. No differences were also reported for exclusive formula fed with either pHF or SF groups, whereas both formula groups showed a higher weight at 4 months as compared to breastfed infants [20].

In the present study, the effect of the feeding regimen on the development of CMPA, AD, and growth parameters within the first 6 months of life was compared between high-risk infants who were exclusively breastfed and those who were formula-fed (exclusively or partially with pHF or SF).

## 2. Materials and Methods

### 2.1. Study Design and Participants

The Allergy Reduction Trial (A.R.T.) is a multicenter, double-blinded, parallel, randomized controlled study assessing differences in the incidence of CMPA and AD in healthy term infants at high risk of developing allergy (infants with a family history of allergy, i.e., past or present asthma, allergic rhinitis/conjunctivitis, AD, food allergy in at least one parent or sibling), as well as differences in growth outcomes within the first six months of life. Participating infants were EBF or randomly allocated to one of the two intervention formulas: (a) a pHF or (b) an SF. These two study formulas produced by FrieslandCampina (Amersfoort, The Netherlands) and provided for free, were similar in macro-nutrients, apart from the protein fraction. In the case of mixed feeding, the required formula intake was at least 40 mL per kg of body weight per day at the age of one month and 60 mL at the age of two months onwards. Allocation to the mixed-feeding group was allowed until the age of 10 weeks.

The study was carried out in Bulgaria, Cyprus, and Greece between 2017–2019, and registered in the Netherlands Trial Registry [Identifier: Trial NL6120 (NTR6259)]. Each study center obtained approval from the respective independent ethics committee.

### 2.2. Recruitment Procedures and Inclusion Criteria

Details of the recruitment procedures and study methodology have been previously described [18]. In brief, families attending maternity clinics during the 7th–9th month of gestation (or shortly after delivery), were interviewed by study researchers to identify those with a family history of allergy. If so, parents were informed about the A.R.T. study and based on their willingness to participate, a pre-consent form was completed. On the day of delivery, parents received detailed information and if all the inclusion criteria were fulfilled, they were asked to decide within four days for their infant’s participation in the study. In case of approval, a consent form was completed and signed.

### 2.3. Follow-Up Evaluation and Compliance

Infants were followed-up bimonthly (2nd, 4th, and 6th month) at the study centers during the first six months of life and additional follow-up was performed at any time point if required (development of any signs of allergy or adverse events). At visits, infants were clinically examined and a questionnaire assessing the presence of CMPA and AD signs or symptoms was applied. The SCORAD and CoMiSS tools were additionally completed. Anthropometric measurements were performed by two well-trained research team members in each center. The calibrated scales SECA 354 with a ±20 g precision below 20 kg were used for assessing body weight, while for body length and head circumference, the infantometer SECA 210 and the non-elastic measuring tapes SECA 211 were used, respectively, measuring to the nearest 0.1 cm. All measurements were performed in triplicates and averaged. In cases where two out of three measurements were different by >100 g for weight, >0.7 cm for length, and >0.5 cm for head circumference, a fourth measurement was performed, and the three nearest measurements were averaged.

Infants allocated to the study formulas had not consumed any other formula (except extensively hydrolyzed formula) from birth. Solid food introduction was allowed after the age of 4 months and no guidance was given regarding the order of introduction. Formula intake was evaluated using a 7-day milk diary completed during the week preceding the 1st, 2nd, 4th, and 6th month of age. Formula consumption of ≥60 mL/kg body weight/day in the 2nd month (and thereafter), was required to assure a formula intake of about 40% of total daily milk intake. The amount of consumed breastmilk in the mixed-feeding groups was estimated using the equation ‘breastmilk (ml) per kg body weight (BW) = (−2.24 × [infant age in weeks) + 164) − (actual intake of formula/kg BW) [21]. Negative values were handled as ‘zero’. For the EBF group, no dietary restrictions were applied to breastfeeding mothers, while infants were exclusively breastfed at least until the age of four months to continue participating in the study.

### 2.4. Definition of Study Outcomes

Detailed information regarding study outcomes has been previously described. In short, CMPA in formula-fed infants was defined as the presence of AD (as below) and/or allergic urticarial rash and/or gastrointestinal manifestations combined with an open positive oral food challenge (OFC) [18].

CMPA in EBF was confirmed by cow’s milk protein (CMP) elimination in the mother’s diet for 7–14 days (depending on the timing of symptoms disappearance) followed by CMP reintroduction in the maternal diet. If symptoms reappeared, then the infant was diagnosed with CMPA.

AD was defined as the clinical diagnosis by the pediatrician accompanied by the recorded scores in SCORAD and CoMiSS tools (total objective score > 1 and Skin Symptoms on Atopic Eczema > 1, respectively) [18].

Z-scores for weight, length, and body mass index (BMI) were calculated and compared with World Health Organization growth charts (“https://www.who.int/tools/child-growth-standards/standards” (accessed on 1 July 2022)).

### 2.5. Statistical Analysis

Sample size calculation for the A.R.T. study has been described previously [18]. Shortly, using a significance error of 5% (2-tailed) and power of 80%, a sample size of 121 infants per treatment arm should be available for evaluation. Assuming a drop-out rate of 30%, 158 infants had to be included per treatment arm.

All statistical analyses were conducted using the SPSS statistical software for Windows (IBM, version 28.0; IBM, Armonk, NY, USA). The normality of the distribution of continuous variables was tested by the Kolmogorov–Smirnov test. Normally distributed continuous variables are presented as means and standard deviations (SD), while non-normally distributed ones are presented as medians and interquartile ranges (IQR). Categorical variables are presented as frequencies (*n*) and percentages (%).

Both per-protocol (PP) and intention–to–treat (ITT) statistical analyses were performed. Between-group differences of continuous variables were tested using either one-way Analysis of Variance (ANOVA) or the non-parametric Kruskal–Wallis test for normally and non-normally distributed variables, respectively. The significance of the association between categorical variables was examined using the chi-squared (χ²) test or the Fisher exact test, whenever appropriate.

The incidence (%), relative risk (RR), and the 95% confidence interval of the RR (95% CI) were calculated for the occurrence of AD and CMPA within the first six months of life. Furthermore, a Poisson General Equation Estimation (GEE) regression analysis was performed to calculate the treatment × time interaction effects on the incidence of AD and CMPA in the pHF or SF groups compared to the EBF group (Model 1). The GEE regression analysis was also stratified for the family history of AD (Model 2) and the amount of breast milk consumed by the mixed-fed infants in the pHF and SF groups (Model 3). In all GEE regression models, adjustments were also made for a wide range of potential confounding factors.

Repeated measures ANOVA was used to examine the between-group differences (treatment effect) in infants’ growth indices (i.e., body weight, length, BMI, and their Z-scores) at baseline, 4 and 6 months of age; the within-group changes (time effect) from baseline to the follow-up time-points in each treatment arm; and the differences among treatment arms in the changes from baseline to the 6-month follow-up (treatment × time interaction effect). Adjustments were made for the potential “confounding effect” of gender, infant’s birth weight, maternal and paternal educational level, region of residence (i.e., urban vs. rural), and the country of the infant’s birth.

All reported *p*-values were two-tailed, and the level of statistical significance was set at *p* < 0.05.

## 3. Results

**Figure 1:**
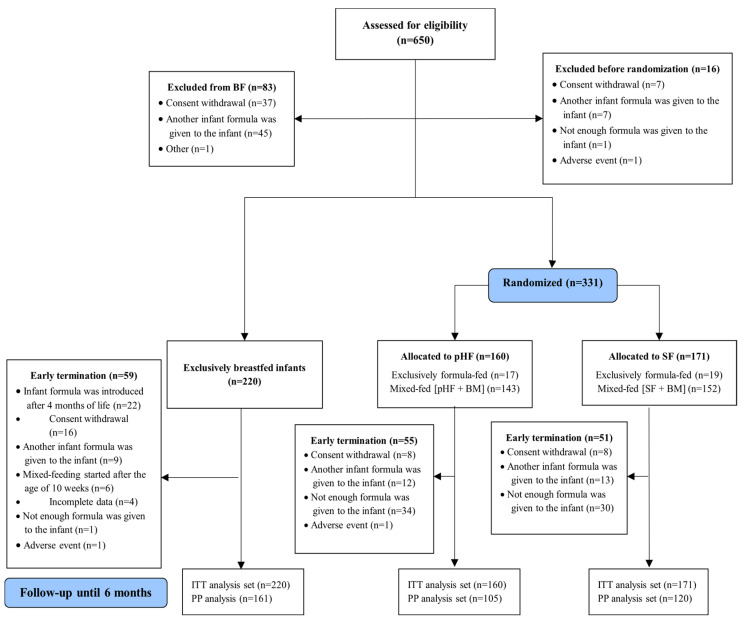
Flow diagram of the A.R.T. population. BF: breastfeeding; pHF: partially hydrolyzed formula; SF: standard formula; PP: Per-Protocol; ITT: intention-to-treat; BM: breast milk.

Of 650 subjects eligible for participating in the study, 99 dropped out before assignment to any group, while 331 infants were randomized to one of the two study formula groups and 220 were exclusively breastfed. The flow diagram of the study population and reasons for dropouts are presented in Figure 1.

### 3.1. Study Populations at Baseline

#### 3.1.1. ITT Population

Table 1 shows that the majority (*n* = 114 out of *n* = 220; 51.8%) of EBF infants were recruited in Greece (*p* < 0.001), whereas formula-fed, or mixed-fed infants were mainly recruited from Cyprus and Bulgaria (*n* = 274 out of *n* = 331; 82.8%, *p* < 0.001). Within countries, Greece recruited more EBF infants as compared to formula or mixed-fed infants, while in Bulgaria this was reversed. In Cyprus, the infants were equally distributed among study groups. Less cesarean deliveries were observed in the EBF group (*p* < 0.001), as compared to pHF and SF groups. In the SF, compared to the EBF group, parents were significantly more often smokers (*p* < 0.038), had a lower educational level (*p* < 0.027), and had a lower contribution of urban families (*p* = 0.008). Early-life infections were defined as viral or bacterial infections occurring during the first 6 months of life.

Regarding anthropometry, head circumference at baseline was different between the study groups (*p* = 0.020), being the smallest in the SF group. At baseline, all infants had negative Z-scores for weight and BMI, with EBF showing the best Z-scores for weight as compared to pHF and SF, and for the BMI Z-score as compared to SF. For length, only the pHF group showed a negative Z-score, although without evident difference among feeding regimens (Table 2).

#### 3.1.2. PP Population

PP population presented similar differences as described for the ITT population (Table 3). Additional differences in the PP population included shorter length at baseline in the SF group compared to EBF, (*p* = 0.029), and a higher number of smoking mothers during pregnancy (*p* = 0.05) (Table 3 and Table 4).

**Figure 2:**
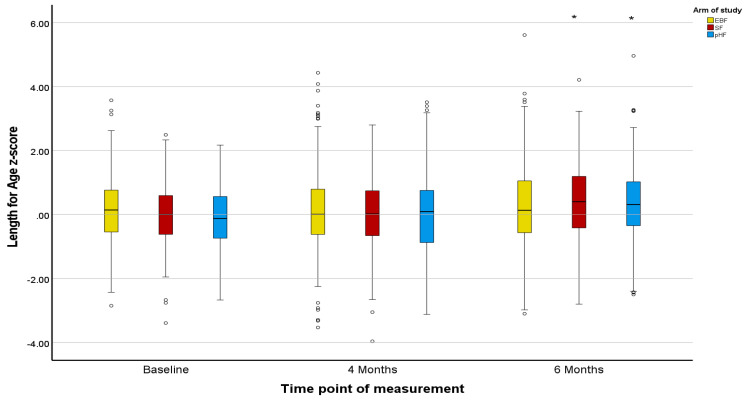
Changes in infants’ length-for-age z-score (LAZ) from baseline to 4 and 6 months of age per treatment arm (ITT analysis). * The asterisks indicate that the changes from baseline to 6 months observed in the SF and pHF arms are significantly higher compared to the relevant change observed in the EBF arm. The dots appearing in the figure represent outlier values.

**Figure 3:**
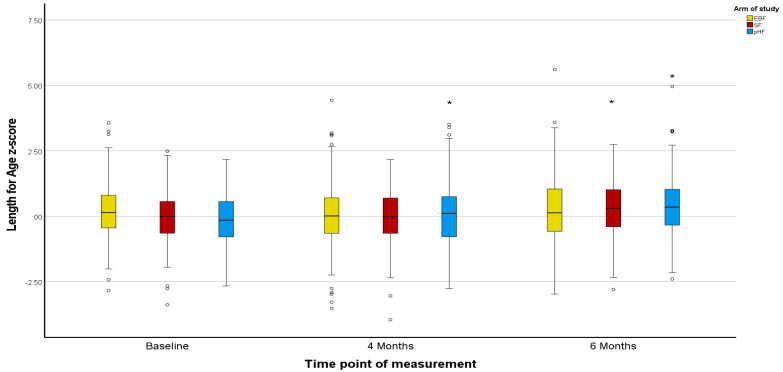
Changes in infants’ length-for-age z-score (LAZ) from baseline to 4 and 6 months of age per treatment arm (PP analysis). * The asterisks indicate that the change from baseline to 4 months observed in the pHF arm is significantly higher compared to the relevant changes observed in the EBF and pHF arms. In addition, the asterisks show that the changes from baseline to 6 months observed in the SF and the pHF arms are significantly higher compared to the relevant change observed in the EBF arm. The dots appearing in the figure represent outlier values.

**Figure 4:**
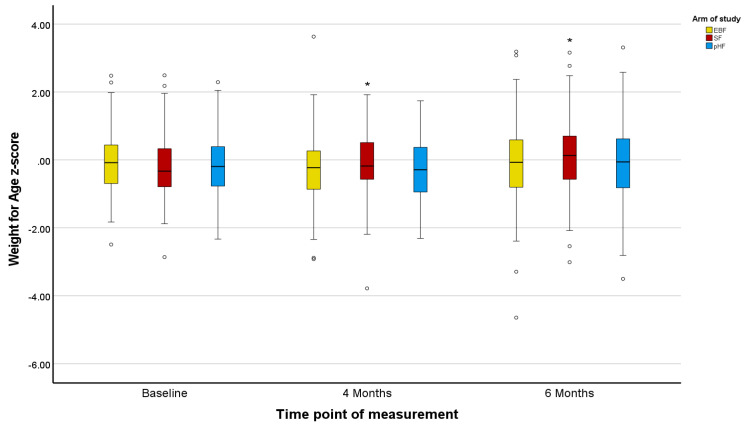
Changes in infants’ weight-for-age z-score (WAZ) from baseline to 4 and 6 months of age per treatment arm (ITT analysis). * The asterisks indicate that the changes from baseline to 4 and 6 months observed in the SF arm are significantly higher compared to the relevant changes observed in the EBF and pHF arms. The dots appearing in the figure represent outlier values.

**Figure 5:**
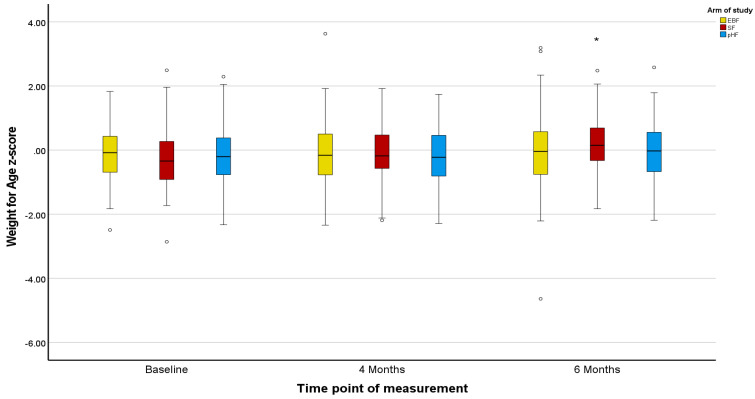
Changes in infants’ weight-for-age z-score (WAZ) from baseline to 4 and 6 months of age per treatment arm (PP analysis). * The asterisks indicate that the changes from baseline to 6 months observed in the SF and pHF arms are significantly higher compared to the relevant change observed in the EBF arm. The dots appearing in the figure represent outlier values.

**Figure 6:**
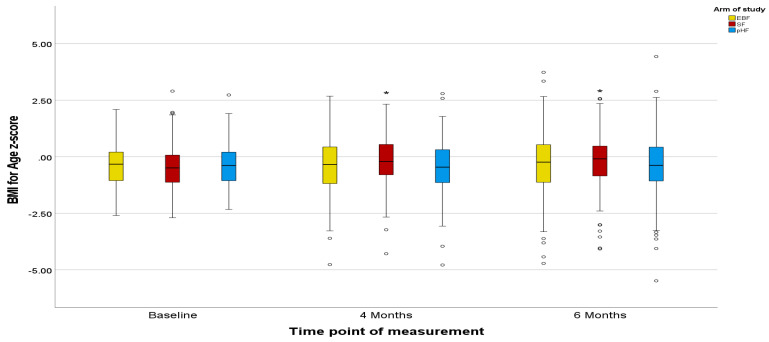
Changes in infants’ BMI-for-age z-score (BAZ) from baseline to 4 and 6 months of age per treatment arm (ITT analysis). * The asterisks indicate that the changes from baseline to 6 months observed in the SF and pHF arms are significantly higher compared to the relevant change observed in the EBF arm. The dots appearing in the figure represent outlier values.

**Figure 7:**
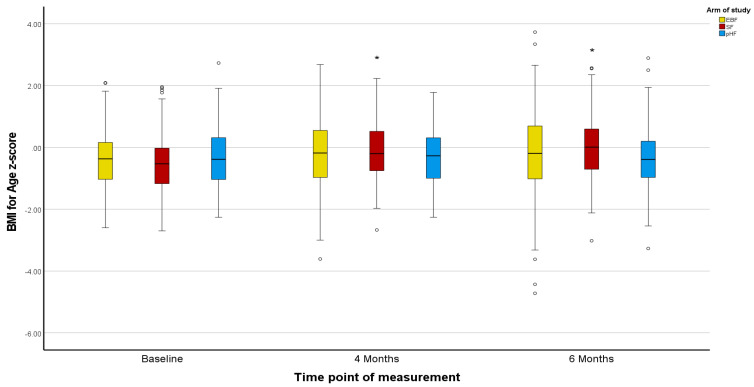
Changes in infants’ BMI-for-age z-score (BAZ) from baseline to 4 and 6 months of age per treatment arm (PP analysis). * The asterisks indicate that the changes from baseline to 4 and 6 months observed in the SF arm are significantly higher compared to the relevant changes observed in the EBF and pHF arms. The dots appearing in the figure represent outlier values.

Z-scores in the PP population agreed with the ITT population, except for marginal differences in the Z-scores for BMI (Figure 2, Figure 3, Figure 4, Figure 5, Figure 6 and Figure 7).

### 3.2. Effect of Intervention

#### 3.2.1. ITT Population

After 6 months of intervention (Table 5), the incidence of AD was significantly higher in the EBF compared to the pHF group, in the presence of a positive family history of AD (95%-CI: 0.09, 0.93, RR: 0.29, *p* = 0.007), while such differences were not present in comparison to the SF group. A trend (*p* = 0.086) towards a lower incidence of CMPA was observed in the pHF compared to the EBF group, whereas no differences were noted among the EBF and SF groups. The reduced incidence of CMPA in the pHF group was present in infants with an estimated breast milk intake above the median daily milk consumption (>278 mL/day) (*p* < 0.001).

The absolute values of body weight, length, head circumference, and BMI increased in all groups during the study, with the SF group showing significantly higher increases in body weight and BMI from baseline to the age of 4 and 6 months, while length changes were more pronounced in the pHF group (Table 2).

The mean Z-scores for length in the formula groups increased during the study, being significant for pHF at 4 and 6 months and for SF at 6 months compared to the baseline. Increases in the length mean Z-score in the EBF group were not significant, and lower as compared to the formula groups (Figure 2). The mean Z-scores for weight initially decreased in EBF (significant) and pHF (not significant) and increased thereafter, with major differences between groups. For the SF group, the mean Z-score for weight increased from baseline onwards (Figure 4). In line with this, the mean Z-score for BMI was different between the groups at ages 4 and 6 months, with SF showing the highest mean Z-score (Figure 6).

#### 3.2.2. PP Population

The protective effect of pHF feeding on AD incidence was also observed in the PP analysis (Table 6).

Absolute body weight, length, head circumference, and BMI increased in all groups during the study, with the BMI in the SF group being higher compared to the pHF 6-month infants (*p* = 0.039). The change in weight, length, and BMI was different between groups at 6 months (Table 4), as was BMI at the age of 4 months. Higher gain for weight and BMI were noted in the SF group, whereas for length in the pHF group.

Z-scores for length were as in the ITT analysis (Figure 2 and Figure 3). For weight, only the mean Z-score in the EBF initially decreased non-significantly at age 4 months but increased thereafter. For the mixed feeding groups, the mean Z-scores for weight increased from baseline onwards, however, such changes were more pronounced in the SF group (Figure 5), in agreement with respective increases in BMI mean Z-scores in the SF group (Figure 7).

### 3.3. The Estimated Breastmilk Intake in Mixed-Fed Groups

These average estimated intakes (for ITT) in quartiles 1–2 and 3–4, at 6 months, were: 0.0 ± 0.0 mL (Q1–Q2, *n* = 93) and 329.5 ± 293.5 (Q3–Q4, *n* = 78) for SF and 0.4 ± 3.4 mL (Q1–Q2, *n* = 80) and 376.3 ± 277.9 (Q3–Q4, *n* = 80) for pHF. For the PP population these values were: 9.3 ± 20.6 mL (Q1–Q2, *n* = 59) and 390.5 ± 305.9 (Q3–Q4, *n* = 59) for SF and 40.4 ± 59.4 mL (Q1–Q2, *n* = 51) and 499.5 ± 271.7 (Q3–Q4, *n* = 50) for pHF. Although the intake of BM in the pHF group tends to be higher than in the SF group, the difference was not significant.

### 3.4. Limitations and Strengths of This Study

Certain limitations must be considered in our study. First, the number of infants included in the final analysis may not be enough to draw firm conclusions. In this respect, although CMPA incidence is lower in the pHF compared to the SF group, differences did not reach statistical significance. Moreover, breastmilk intake is estimated using an equation, in which the expected breastmilk intake per kg of body weight (BW) is calculated based on the age of the infant. Therefore, in the absence of data concerning the exact given amount of breast milk per kg of BW in each participant of the three different feeding patterns, this method is only a crude estimation of breast milk intake, that may be subjected to biased correlations. Nevertheless, the double-blinded randomized controlled design of the study, the assessment of allergy and anthropometric outcomes in infants by specialized well-trained study members, the objectively confirmed CMPA symptoms, and the statistical analysis performed by a third independent collaborator are important strengths of the present study.

## 4. Discussion

The present study indicates that infants at high risk for allergy (based on a family history of allergy) may benefit from a combination of breastmilk and the studied infant formula with partially hydrolyzed whey protein. This combination resulted in a lower incidence of AD in a subpopulation of infants with a positive family history for AD, in both ITT and PP populations. A trend was found towards a lower incidence of CMPA, including food challenge confirmed cases, in the pHF group, as compared to EBF, in both the ITT and PP data sets. The incidences of AD and CMPA were similar between the EBF and SF groups. With respect to growth, all groups showed small, negative mean Z-scores for body weight and BMI at baseline. Throughout the study growth in all groups complied with WHO standards, with the SF group showing the highest increase in body weight and BMI, while length increased the most in the pHF group. Z-scores for weight in the EBF and pHF groups were close together and developed similarly throughout the study, with an initial decrease at the age of 4 months in the EBF group and an increase thereafter.

The protective effect on certain allergy outcomes in the pHF group was mainly observed in the mixed-fed infants, which represent the vast majority of the pHF group in our cohort. The number of exclusively formula-fed infants was too small to draw any conclusion. In line with our results, the GINI study also showed the protective effect of a partially hydrolyzed formula on allergic manifestations, i.e., atopic dermatitis [22], whilst review studies even in the general population are indicative of the protective effect of pHF in non-exclusively breastfed infants [19].

Studies on the role of early supplementation of intact CMP on the later development of allergy-associated diseases are indicative of a protective effect, depending however on the timing of CMP formula introduction. Early CMP introduction, but not earlier than two weeks of life, has been associated with a lower risk for IgE-mediated CMPA [23], while early as three days of life supplementation followed by complete CMP avoidance may result in opposite effects [16,24]. Even more, the timing for commencing pHF consumption is considered as important since the most beneficial effect with respect to allergy prevention is observed during the first 6 months of life [25]. It might be that EBF from birth onwards may supply too much allergenic B-lactoglobulin, whereas after an initial short period of ‘no allergens’ (extensively hydrolyzed formula), a minimum number of allergens is necessary to build tolerance. Moreover, the minimum allergenicity albeit combined with residual antigenicity of proteins contained in the partially hydrolyzed formulas, might facilitate tolerance at least in a subpopulation of high risk for allergy infants [26].

The lower incidence of AD in the pHF group was noted in infants with a positive family history of AD. Atopic Dermatitis in the core family has been long considered a significant risk factor for the development of any allergy-associated disease in the offspring, compared to any other allergic disease, as shown by epidemiological, intervention, and genetic studies [22,27]. It is plausible, that the beneficial effect of the pHF compared to the SF group on AD and perhaps on CMPA outcomes depends highly on the genetic background, which potentially modifies the preventive effect of a hydrolysate, as was previously suggested [22]. Moreover, it is not anticipated that the country of origin of recruited babies imposes a role in the occurrence of allergy-associated outcomes, since food allergy incidence is low in the participating centers, as was previously shown [28,29]. In our cohort, the beneficial effect on allergy outcomes was more pronounced in infants receiving certain amounts of breast milk, although exact estimations could not be determined. Differences in the prevalence of breastfeeding included infants could be attributed to the different maternity hospitals’ practices, since in Greece the majority of participating centers are officially labeled as “Baby Friendly Hospitals”. In accordance, previous reports on CMP supplementation have highlighted the significance of amounts of breast milk consumed on the development of CMPA, suggesting that even small amounts of breast milk can provide a beneficial effect [24], as was in our cohort in the pHF group. It is plausible that a beneficial effect of pHF could explain the lower incidence of CMPA events in infants with higher intakes of breast milk. The amount of breast milk in the pHF group might be linearly related to a lower incidence of CMPA. A high percentage of breast milk contains cow’s milk-derived peptides in small amounts [30], which may induce tolerance.

With respect to growth outcomes, although increases in body weight and BMI mean Z-scores from baseline to 4 and 6 months were more pronounced for the SF group, respective length increases were significantly higher in the pHF group. It has been previously documented that the specific whey-based pHF formula used in our study, is non-inferior regarding all infant growth outcomes compared to SF, although a margin of −3 g/day was noted in a three-month intervention period for the pHF compared to the SF group [31], while review reports are confirmatory of the normal growth in infants consuming pHFs [19]. Infants fed with pHF presented equivalent growth to those fed with SF, while a non-inferiority study showed that mixed-fed (pHF and breastfed) closely tracked EBF infants [19,20].

## 5. Conclusions

The data from our study support that infants at high risk for allergy who are not exclusively breastfed, benefit with regards to allergy-associated outcomes, when supplemented with a specific whey-based pHF complementary to breastfeeding compared to mixed-feeding with a standard formula of intact protein. Supplementation of the studied pHF to BM resulted in reduced incidence of atopic dermatitis and CMPA, particularly in those high risk for allergy infants with a family history of atopic dermatitis. The growth outcomes of study participants during the first 6 months of life were within the normal range in all feeding regimens. The findings of the A.R.T. study suggest the use of this specific whey-based pHF in mixed-fed infants for the prevention of allergy outcomes within the first six months of life.

## Figures and Tables

**Table 1:** Descriptive characteristics of study participants allocated to the three treatment arms at baseline (ITT analysis dataset).

	ITT Analysis Dataset				
	EBF Group (*N* = 220)	pHF Group (*N* = 160)	SF Group (*N* = 171)	*p*-Value	Total Sample (*N* = 551)
Infant’s characteristics					
Country of infant’s birth					
Bulgaria, *n* (%)	48 (31.8) ^a,b^	76 (47.5) ^a^	82 (48.0) ^b^	**<0.001**	206 (37.4)
Cyprus, *n* (%)	58 (26.4)	55 (34.4)	61 (35.7)		174 (31.6)
Greece, *n* (%)	114 (51.8) ^a,b^	29 (18.1) ^a^	28 (16.4) ^b^		171 (31.0)
Normal conception, *n* (%)	215 (98.2) ^a^	148 (93.1) ^a^	164 (95.9)	**0.045**	527 (96.0)
Gestational age, weeks, mean (SD)	38.9 (1.0) ^a^	38.6 (1.2) ^a^	38.7 (1.0)	**0.025**	38.8 (1.1)
Cesarean delivery, *n* (%)	101 (45.9) ^a,b^	106 (66.3) ^a^	106 (62.0) ^b^	**<0.001**	313 (56.8)
Birth weight, g, mean (SD)	3303.6 (392.3)	3270.5 (433.6)	3278.1 (453.7)	0.722	3286.1 (423.6)
Head circumference, cm, mean (SD)	34.4 (1.2)	34.2 (1.2)	34.1 (1.1)	**0.011**	34.2 (1.2)
Gender, female, *n* (%)	104 (47.3)	67 (41.9)	78 (45.6)	0.575	249 (45.2)
Mother’s characteristics					
Age, years, mean (SD)	32.5 (4.9)	31.7 (5.1)	31.3 (5.1)	0.062	31.9 (5.1)
Educational level					
≤14 years, *n* (%)	60 (27.3) ^b^	60 (37.5)	70 (40.9) ^b^	**0.012**	190 (34.5)
>14 years, *n* (%)	160 (72.7) ^b^	100 (62.5)	101 (59.1) ^b^		361 (65.5)
Mother smoking during pregnancy, *n* (%)	13 (5.9)	15 (9.4)	22 (12.9)	0.059	50 (9.1)
Mother smoking at baseline, *n* (%)	25 (11.4) ^b^	28 (17.5)	41 (24.0) ^b^	**0.004**	94 (17.1)
Father’s characteristics					
Age, years, mean (SD)	35.1 (5.6)	34.5 (5.7)	34.1 (5.6)	0.210	34.6 (5.6)
Educational level					
≤14 years	92 (41.8) ^b^	76 (47.8)	95 (55.6) ^b^	**0.026**	263 (47.8)
>14 years	128 (58.2) ^b^	83 (52.2)	76 (44.4) ^b^		287 (52.2)
Father smoking at baseline, *n* (%)	82 (37.3) ^b^	77 (48.1)	83 (48.5) ^b^	**0.037**	242 (43.9)
Family characteristics					
Family members at home, median, IQR	4.0 (1.0)	3.0 (1.0)	3.0 (1.0)	0.329	3.0 (1.0)
Urban residence, *n* (%)	203 (92.3) ^b^	146 (91.3)	141 (82.9) ^b^	**0.008**	490 (89.1)
Presence of pets indoors at home, *n* (%)	39 (17.7)	32 (20.0)	38 (22.2)	0.207	109 (19.8)
Medical history					
Family history of:					
Allergic asthma, *n* (%)	66 (30.0)	42 (26.3)	42 (24.6)	0.462	150 (27.2)
Rhinitis, *n* (%)	130 (59.1)	81 (50.6)	85 (49.7)	0.118	296 (53.7)
Atopic dermatitis, *n* (%)	75 (34.1)	46 (28.7)	44 (25.7)	0.186	165 (29.9)
Urticaria, *n* (%)	32 (14.6)	22 (13.8)	28 (16.4)	0.789	82 (14.9)
Food allergy, *n* (%)	70 (31.8)	45 (28.1)	52 (30.4)	0.741	167 (30.3)
Occurrence of early life infections in infants					
No infections, *n* (%)	176 (80.0)	114 (71.3)	127 (74.3)	0.177	417 (75.7)
Before 1st month, *n* (%)	7 (3.2)	3 (1.9)	6. (3.5)		16 (2.9)
After 1st month, *n* (%)	37 (16.8)	43 (26.9)	38 (22.2)		118 (21.4)

EBF: exclusive breastfeeding; pHF: partially hydrolyzed formula; SF:standard formula; ITT: Intention-to-treat; *N*: Number of study participants; *n*: number of non-missing observations; SD: Standard Deviation; IQR: Interquartile Range. *p*-values for the comparison of categorical variables derived from the chi-square test or the Fisher exact test, whenever appropriate. *p*-values for the comparison of continuous variables derived from one-way ANOVA or the Kruskal Wallis test for normally and non-normally distributed variables respectively. All *p*-values in bold indicate statistically significant differences among treatment arms. Percentages sharing the same superscript letter within the same raw are statistically significantly different between them, according to pairwise comparisons using the Bonferroni correction to account for type I error.

**Table 2:** Changes in infants’ growth indices from baseline to 4 and 6 months of age per treatment arm (ITT analysis).

	Time-Point of Evaluation (ITT Analysis Dataset)			Time Effect (4-Month Change)	Time Effect (6-Month Change)
	Baseline	Visit 2 (4 Months)	Visit 3 (6 Months)		
	Mean (SEM)	Mean (SEM)	Mean (SEM)	Mean Change (95% CI)	Mean Change (95% CI)
Body weight (kg)					
EBF group (*n* = 219)	3.25 (0.007) ^a,b^	6.62 (0.046)	7.65 (0.060)	**3.37 (3.28; 3.46)**	**4.41 (4.29; 4.53)**
pHF group (*n* = 159)	3.22 (0.008) ^a^	6.63 (0.052)	7.68 (0.069)	**3.41 (3.31; 3.51)**	**4.47 (4.33; 4.60)**
SFgroup (*n* = 170)	3.20 (0.008) ^b^	6.76 (0.052)	7.82 (0.068)	**3.55 (3.45; 3.66)**	**4.62 (4.49; 4.75)**
Treatment effect (*p*-value) *	**<0.001**	0.085	0.148	**0.025**	**0.017**
Length (cm)					
EBF group (*n* = 219)	49.9 (0.09)	63.4 (0.17)	67.4 (0.19)	**13.4 (13.1; 13.7)**	**17.5 (17.1; 17.8)**
pHF group (*n* = 159)	49.8 (0.11)	63.6 (0.19)	67.9 (0.21)	**13.8 (13.4; 14.1)**	**18.1 (17.7; 18.5)**
SFgroup (*n* = 170)	50.0 (0.10)	63.4 (0.19)	67.9 (0.21)	**13.5 (13.1; 13.8)**	**17.9 (17.6; 18.3)**
Between-group effect (*p*-value) *	0.412	0.770	0.127	0.305	0.056
Body Mass Index (Kg/m^2^)					
EBF group (*n* = 219)	13.0 (0.05) ^b^	16.5 (0.11)	16.8 (0.12)	**3.5 (3.2; 3.7)**	**3.8 (3.6; 4.1)**
pHF group (*n* = 159)	12.9 (0.06)	16.4 (0.13)	16.7 (0.14)	**3.5 (3.2; 3.7)**	**3.7 (3.5; 4.0)**
SFgroup (*n* = 170)	12.8 (0.06) ^b^	16.8 (0.13)	16.9 (0.14)	**4.0 (3.7; 4.3)**	**4.2 (3.9; 4.4)**
Between-group effect (*p*-value) *	**0.035**	0.075	0.405	**0.004**	**0.004**
Head Circumference (cm)					
EBF group (*n* = 219)	34.4 (0.07) ^b^	41,3 (0.08)	43.0 (0.10)	**6.9 (6.8; 7.1)**	**8.6 (8.5; 8.8)**
pHF group (*n* = 159)	34.2 (0.07)	41.3 (0.10)	42.9 (0.11)	**7.1 (6.9; 7.3)**	**8.7 (8.5; 8.9)**
SFgroup (*n* = 170)	34.1 (0.07) ^b^	41.3 (0.09)	43.0 (0.11)	**7.2 (7.0; 7.4)**	**8.9 (8.7; 9.1)**
Between-group effect (*p*-value) *	**0.020**	0.997	0.907	0.083	0.057
Weight-for-age Z-score					
EBF group (*n* = 219)	−0.13 (0.02) ^a,b^	−0.28 (0.06)	−0.13 (0.07)	**−0.15 (−0.27; −0.03)**	0.00 (−0.14; 0.14)
pHF group (*n* = 159)	−0.19 (0.02) ^a^	−0.25 (0.07)	−0.08 (0.08)	−0.06 (−0.19; 0.08)	0.12 (−0.04; 0.27)
SFgroup (*n* = 170)	−0.22 (0.02) ^b^	−0.08 (0.07)	0.09 (0.08)	**0.14 (0.009; 0.27)**	**0.31 (0.16; 0.46)**
Treatment effect (*p*-value) *	**<0.001**	0.069	0.109	**0.006**	**0.003**
Length-for-age-Z-score					
EBF group (*n* = 219)	0.04 (0.05)	0.04 (0.08)	0.19 (0.08)	0.002 (−0.14; 0.15)	0.15 (−0.01; 0.31)
pHF group (*n* = 159)	−0.06 (0.06)	0.14 (0.09)	0.43 (0.10)	**0.19 (0.03; 0.36)**	**0.48 (0.30; 0.67)**
SFgroup (*n* = 170)	0.03 (0.06)	0.08 (0.09)	0.44 (0.09)	0.04 (−0.12; 0.21)	**0.41 (0.23; 0.59)**
Between-group effect (*p*-value) *	0.419	0.722	0.081	0.206	**0.019**
Body Mass Index-for age Z-score					
EBF group (*n* = 219)	−0.34 (0.04) ^b^	−0.40 (0.04)	−0.31 (0.09)	−0.06 (−0.23; 0.10)	0.03 (−0.15; 0.21)
pHF group (*n* = 159)	−0.37 (0.05)	−0.43 (0.09)	−0.42 (0.10) ^c^	−0.06 (−0.25; 0.13)	−0.05 (−0.26; 0.15)
SFgroup (*n* = 170)	−0.50 (0.05) ^b^	−0.16 (0.09)	−0.22 (0.10) ^c^	**0.34 (0.15; 0.52)**	**0.29 (0.09; 0.49)**
Between-group effect (*p*-value) *	**0.032**	0.069	0.333	**0.003**	**0.002**

SEM: Standard Error of Mean; EBF: exclusive breastfeeding; pHF: partially hydrolyzed formula; SF: standard formula; ITT: intention-to-treat. *All *p*-values derived from the Analysis of Variance for Repeated Measures. All *p*-values in bold indicate statistically significant between-group differences among treatment arms, while mean changes in bold indicate within-group changes from baseline to 6 months. Mean values sharing the same superscript letter (i.e., ^a, b^ or ^c^) indicate statistically significant differences between treatment arms in the relevant pairwise comparisons. All statistical analyses were adjusted for the potential confounding effect of gender, infant’s birth weight, maternal and paternal educational level, region of residence (i.e., urban vs. rural), and the country of infant’s birth.

**Table 3:** Descriptive characteristics of study participants allocated to the three treatment arms at baseline (PP analysis dataset).

	EBF Group (*N* = 161)	pHF Group (*N* = 105)	SF Group (*N* = 120)	*p*-Value	Total Sample (*N* = 386)
Infant’s characteristics					
Country of infant’s birth					
Bulgaria, *n* (%)	35 (21.7) ^a,b^	45 (42.9) ^a^	58 (48.3) ^b^	**<0.001**	138 (35.8)
Cyprus, *n* (%)	33 (20.5) ^a,b^	45 (42.9) ^a^	43 (35.8) ^b^		121 (31.3)
Greece, *n* (%)	93 (57.8) ^a,b^	15 (14.3) ^a^	19 (15.8) ^b^		127 (32.9)
Normal conception, *n* (%)	157 (97.5)	97 (92.4)	113 (94.2)	0.214	367 (95.3)
Gestational age, weeks, mean (SD)	38.9 (1.0) ^a^	38.6 (1.2) ^a^	38.7 (1.0)	**0.023**	38.8 (1.1)
Cesarean delivery, *n* (%)	72 (44.7) ^a,b^	69 (65.7) ^a^	73 (60.8) ^b^	**0.001**	214 (55.4)
Birth weight, g, mean (SD)	3290.1 (374.3)	3257.1 (431.4)	3246.7 (449.6)	0.656	3267.6 (413.9)
Weight at baseline, g, mean (SD)	3248.8 (378.3)	3198.5 (409.8)	3171.0 (430.9)	0.263	3210.9 (404.2)
Length at baseline, cm, mean (SD)	50.2 (2.0) ^a^	49.5 (1.8) ^a^	49.8 (2.0)	**0.029**	49.9 (2.0)
Head circumference, cm, mean (SD)	34.4 (1.2)	34.1 (1.1)	34.1 (1.1)	**0.103**	34.2 (1.2)
Gender, female, *n* (%)	77 (47.8)	47 (44.8)	55 (45.8)	0.878	179 (46.4)
Mother’s characteristics					
Age, years, mean (SD)	32.9 (4.7) ^a,b^	31.3 (4.8) ^a^	31.4 (5.4) ^b^	**0.010**	32.0 (5.0)
Educational level					
≤14 years, *n* (%)	44 (27.3) ^b^	43 (41.0)	52 (43.3) ^b^	**0.010**	139 (36.0)
>14 years, *n* (%)	117 (72.7) ^b^	62 (59.0)	68 (56.7) ^b^		247 (64.0)
Mother smoking during pregnancy, *n* (%)	10 (6.2) ^b^	10 (9.5)	18 (15.0) ^b^	**0.050**	38 (9.8)
Mother smoking at baseline, *n* (%)	17 (10.6) ^b^	20 (19.0)	29 (24.2) ^b^	**0.009**	66 (17.1)
Father’s characteristics					
Age, years, mean (SD)	35.5 (5.5)	34.4 (6.0)	34.1 (5.7)	0.099	34.8 (5.7)
Educational level					
≤14 years, *n* (%)	64 (39.8) ^b^	53 (51.0)	67 (55.8) ^b^	**0.021**	184 (47.8)
>14 years, *n* (%)	97 (60.2) ^b^	52 (49.0)	53 (44.2) ^b^		201 (52.2)
Father smoking at baseline, *n* (%)	61 (37.9)	55 (52.4)	54 (45.0)	0.065	170 (44.0)
Family characteristics					
Family members at home, median, IQR	3.0 (1.0)	3.0 (1.0)	3.5 (1.0)	0.821	3.0 (1.0)
Urban residence, *n* (%)	150 (93.2)	96 (91.4)	102 (85.7)	0.103	348 (90.4)
Presence of pets indoors at home, *n* (%)	29 (18.0)	25 (23.8)	28 (23.3)	0.699	82 (21.2)
Medical history					
Family history of:					
Allergic asthma, *n* (%)	53 (32.9)	25 (23.8)	30 (25.0)	0.184	108 (28.0)
Rhinitis, *n* (%)	96 (59.6)	52 (49.5)	59 (49.2)	0.135	207 (53.6)
Atopic dermatitis, *n* (%)	59 (36.6)	26 (24.8)	34 (28.3)	0.095	119 (30.8)
Urticaria, *n* (%)	28 (17.4)	15 (14.3)	21 (17.5)	0.760	64 (16.6)
Food allergy, *n* (%)	47 (29.2)	31 (29.5)	33 (27.5)	0.934	111 (28.8)
Occurrence of early life infections in infants					
No infections, *n* (%)	126 (78.3) ^a^	63 (60.0) ^a^	79 (65.8)	**0.005**	368 (69.4)
Before 1st month, *n* (%)	6 (3.7)	2 (1.9)	6 (5.0)		14 (3.6)
After 1st month, *n* (%)	29 (18.0) ^a^	40 (38.1) ^a^	35 (29.2)		104 (26.9)

EBF: exclusive breastfeeding; pHF: partially hydrolysed formula; SF: Standard formula; PP: Per-protocol; N: Number of study participants; n: number of non-missing observations; SD: Standard Deviation; IQR: Interquartile Range. *P*-values for the comparison of categorical variables derived from the chi-square test or the Fisher exact test, whenever appropriate. *P*-values for the comparison of continuous variables derived from one-way ANOVA or the Kruskal Wallis test for normally and non-normally distributed variables respectively. All *P*-values in bold indicate statistically significant differences among treatment arms. Percentages sharing the same superscript letter (i.e., ^a^ or ^b^) within the same raw are statistically significantly different between them, according to pairwise comparisons using the Bonferroni correction to account for type I error.

**Table 4:** Changes in infants’ growth indices from baseline to 4 and 6 months of age per treatment arm (PP analysis).

	Time-Point of Evaluation (ITT Analysis Dataset)			Time Effect (4-Month Change)	Time Effect (6-Month Change)
	Baseline	Visit 2 (4 Months)	Visit 3 (6 Months)		
	Mean (SEM)	Mean (SEM)	Mean (SEM)	Mean Change (95% CI)	Mean Change (95% CI)
Body weight (kg)					
EBF group (*n* = 160)	3.23 (0.008) ^a,b^	6.70 (0.054)	7.67 (0.067)	**3.47 (3.36; 3.57)**	**4.43 (4.30; 4.57)**
pHF group (*n* = 104)	3.20 (0.009) ^a^	6.68 (0.065)	7.66 (0.081)	**3.48 (3.35; 3.60)**	**4.46 (4.30; 4.62)**
SF group (*n* = 119)	3.18 (0.009) ^b^	6.77 (0.060)	7.87 (0.075)	**3.59 (3.47; 3.71)**	**4.69 (4.54; 4.84)**
Treatment effect (*p*-value) *	**<0.001**	0.489	0.073	0.260	**0.029**
Length (cm)					
EBF group (*n* = 160)	49.9 (0.12)	63.2 (0.19)	67.3 (0.22)	**13.2 (12.9; 13.6)**	**17.4 (16.9; 17.8)**
pHF group (*n* = 104)	49.7 (0.14)	63.6 (0.23)	68.0 (0.26)	**13.9 (13.5; 14.3)**	**18.3 (17.8; 18.8)**
SF group (*n* = 119)	49.9 (0.13)	63.4 (0.21)	67.7 (0.25)	**13.5 (13.1; 13.9)**	**17.8 (17.3; 18.2)**
Between-group effect (*p*-value) *	0.408	0.321	0.124	0.055	**0.013**
Body Mass Index (Kg/m^2^)					
EBF group (*n* = 160)	12.9 (0.07)	16.8 (0.12)	16.9 (0.14)	**3.8 (3.6; 4.1)**	**4.0 (3.7; 4.3)**
pHF group (*n* = 104)	12.9 (0.08)	16.5 (0.15)	16.6 (0.17) ^c^	**3.5 (3.2; 3.9)**	**3.7 (3.3; 4.0)**
SF group (*n* = 119)	12.7 (0.07)	16.8 (0.14)	17.2 (0.16) ^c^	**4.1 (3.8; 4.4)**	**4.4 (4.1; 4.8)**
Between-group effect (*p*-value) *	0.053	0.136	**0.039**	**0.029**	**0.004**
Head Circumference (cm)					
EBF group (*n* = 160)	34.3 (0.08)	41.3 (0.10)	42.9 (0.11)	**7.0 (6.8; 7.2)**	**8.7 (8.5; 8.9)**
pHF group (*n* = 104)	34.1 (0.09)	41.1 (0.12)	42.9 (0.13)	**7.0 (6.8; 7.2)**	**8.8 (8.5; 9.0)**
SF group (*n* = 119)	34.1 (0.09)	41.3 (0.11)	43.0 (0.12)	**7.2 (7.0; 7.4)**	**8.9 (8.7; 9.1)**
Between-group effect (*p*-value) *	0.316	0.384	0.845	0.249	0.247
Weight-for-age z-score					
EBF group (*n* = 160)	−0.15 (0.02) ^a,b^	−0.17 (0.09)	−0.09 (0.08) ^b^	−0.02 (−0.16; 0.12)	0.06 (−0.09; 0.22)
pHF group (*n* = 104)	−0.23 (0.02) ^a^	−0.18 (0.08)	−0.08 (0.09) ^c^	0.05 (−0.12; 0.22)	0.15 (−0.03; 0.33)
SF group (*n* = 119)	−0.26 (0.02) ^b^	−0.05 (0.08)	0.17 (0.08) ^b,c^	**0.21 (0.06; 0.37)**	**0.43 (0.26; 0.60)**
Treatment effect (*p*-value) *	**0.001**	0.409	**0.048**	0.094	**0.006**
Length-for-age-z-score					
EBF group (*n* = 160)	0.04 (0.06)	−0.05 (0.09)	0.15 (0.10)	−0.09 (−0.26; 0.08)	0.11 (−0.08; 0.30)
pHF group (*n* = 104)	−0.08 (0.08)	0.19 (0.11)	0.49 (0.12)	**0.27 (0.07; 0.47)**	**0.57 (0.34; 0.79)**
SF group (*n* = 119)	0.03 (0.07)	0.08 (0.10)	0.36 (0.11)	0.06 (−0.13; 0.25)	**0.34 (0.12; 0.55)**
Between-group effect (*p*-value) *	0.448	0.256	0.099	**0.033**	**0.007**
Body Mass Index-for age z-score					
EBF group (*n* = 160)	−0.37 (0.05)	−0.18 (0.08)	−0.23 (0.10)	**0.19 (0.002; 0.37)**	0.14 (−0.07; 0.35)
pHF group (*n* = 104)	−0.39 (0.06)	−0.37 (0.10)	−0.46 (0.12)^c^	0.02 (−0.20; 0.24)	−0.07 (−0.32; 0.18)
SF group (*n* = 119)	−0.55 (0.06)	−0.13 (0.09)	−0.05 (0.11)^c^	**0.43 (0.22; 0.63)**	**0.50 (0.26; 0.73)**
Between-group effect (*p*-value) *	0.052	0.171	**0.041**	**0.026**	**0.003**

SEM: Standard Error of Mean; EBF: exclusive breastfeeding; pHF: partially hydrolyzed formula; SF: standard formula; PP: Per Protocol. *All *p*-values derived from the Analysis of Variance for Repeated Measures. All *p*-values in bold indicate statistically significant between-group differences among treatment arms, while mean changes in bold indicate within-group changes from baseline to 6 months. Mean values sharing the same superscript letter indicate significant differences between treatment arms in the relevant pairwise comparisons. All statistical analyses were adjusted for the potential confounding effect of gender, infant’s birth weight, maternal and paternal educational level, region of residence (i.e., urban vs. rural), and the country of infant’s birth.

**Table 5:** Incidence and relative risk for CMPA and AD within the first six months of life in exclusively breastfed, exclusively formula-fed, and mixed-fed infants in partially hydrolyzed and standard formula groups (ITT analysis dataset).

	Treatment Arms			RR_1_ (95% CI) (PHF/ExcBF)	*p*-Value_1_	RR_2_ (95% CI) (SF/ExcBF)	*p*-Value_2_	*p*-Value_3_
	EBF	pHF	SF					
Model 1	(*N* = 220)	(*N* = 160)	(*N* = 171)					
AD, *n* (%)	38 (17.3)	17 (10.6)	32 (18.7)	0.62 (0.36, 1.05)	0.064	1.08 (0.71, 1.66)	0.709	0.069
CMPA, *n* (%)	21 (9.5)	8 (5.0)	16 (9.4)	0.52 (0.24, 1.15)	0.086	0.98 (0.53, 1.82)	0.953	0.154
Model 2								
FHAD (+)	(*N* = 75)	(*N* = 46)	(*N* = 44)					
AD, *n* (%)	17 (22.7)	3 (6.5)	12 (27.3)	0.29 (0.09, 0.93)	0.007	1.20 (0.63, 2.28)	0.576	0.003
CMPA, *n* (%)	12 (16.0)	3 (6.5)	7 (15.9)	0.41 (0.12, 1.37)	0.088	0.99 (0.42, 2.34)	0.990	0.166
FHAD (−)	(*N* = 145)	(*N* = 114)	(*N* = 127)					
AD, *n* (%)	21 (14.5)	14 (12.3)	20 (15.7)	0.85 (0.45, 1.59)	0.628	1.08 (0.62, 1.91)	0.769	0.751
CMPA, *n* (%)	9 (6.2)	5 (4.4)	9 (7.1)	0.71 (0.24, 2.05)	0.525	1.14 (0.47, 2.79)	0.770	0.651
Model 3								
Lower % BM intake	(*N* = 0)	(*N* = 126)	(*N* = 150)					
AD, *n* (%)	N/A	13 (10.3)	28 (18.7)	N/A	N/A	N/A	N/A	N/A
CMPA, *n* (%)	N/A	8 (6.3)	15 (10.0)	N/A	N/A	N/A	N/A	N/A
Higher % BM intake	(*N* = 220)	(*N* = 34)	(*N* = 21)					
AD, *n* (%)	38 (17.3)	4 (11.8)	4 (19.0)	0.68 (0.26, 1.79)	0.367	1.10 (0.44, 2.79)	0.849	0.636
CMPA, *n* (%)	21 (9.5)	0 (0.0)	1 (4.8)	N/A	<0.001	0.50 (0.07, 3.53)	0.347	<0.001

AD: atopic dermatitis; CMPA: cow’s milk protein allergy, confirmed by oral food challenge; EBF: exclusive breastfeeding; pHF: partially hydrolyzed formula; SF: standard formula; ITT: intention-to-treat; *N*: number of study participants; RR_1_: Relative risk for CMPA or AD EBF vs. pHF; RR_2_: Relative risk for CMPA or AD EBF vs. SF; CI: Confidence Interval; FHAD (+): family history of AD; FHAD (−): no family history of AD; Lower % BM intake: Percentage consumption of breast milk in the mixed-fed infants that is lower than or equal to the median average daily milk consumption (i.e., ≤41.8% of total milk consumption coming from breast milk or ≤278 mL of breast milk); Higher % BM intake: Percentage consumption of breast milk in the mixed-fed infants that is higher than the median average daily milk consumption (i.e., >41.8% of total milk consumption coming from breast milk or >278 mL of breast milk). Model 1 was adjusted for the potential confounding effect of gender, type of conception (i.e., normal vs. IVF), gestational age, type of delivery (i.e., labor vs. cesarean), the amount of breast milk consumed by infants, the occurrence of early life infections, maternal and paternal educational level, maternal and paternal smoking at home, the presence of pets at home, the region of residence (i.e., urban vs. rural) and the country of infant’s birth. The rest of the models presented in the table were further adjusted for the interaction between treatment arm and FHAD (Model 2), and the amount of Breast Milk Intake by infants (Model 3). All *p*-values derived from Poisson Generalized Estimating Equation (GEE) regression analysis. *p*-value_1_ indicates the statistical significance of the treatment effect in the pHF compared to the EBF group; *p*-value_2_ indicates the statistical significance of the treatment effect in the SF compared to the EBF group; *p*-value_3_ indicates the statistical significance of the overall treatment x time effect when comparing all treatment arms.

**Table 6:** Incidence and relative risk for CMPA and AD within the first six months of life in exclusively breastfed, exclusively formula-fed, and mixed-fed infants (PP analysis dataset).

	Treatment Arms			RR_1_ (95% CI) (PHF/ExcBF)	*p*-Value_1_	RR_2_ (95% CI) (SF/ExcBF)	*p*-Value_2_	*p*-Value_3_
	EBF	pHF	SF					
Model 1	(*N* = 161)	(*N* = 105)	(*N* = 120)					
AD, *n* (%)	33 (19.9)	12 (11.4)	29 (24.2)	0.58 (0.31, 1.07)	0.066	1.22 (0.78, 1.89)	0.371	0.031
CMPA, *n* (%)	21 (13.0)	7 (6.7)	14 (11.7)	0.51 (0.23, 1.16)	0.085	0.89 (0.48, 1.69)	0.748	0.195
Model 2								
FHAD (+)	(*N* = 59)	(*N* = 26)	(*N* = 34)					
AD, *n* (%)	14 (23.7)	2 (7.7)	10 (29.4)	0.32 (0.08, 1.33)	0.035	1.24 (0.62, 2.48)	0.550	0.031
CMPA, *n* (%)	12 (20.3)	3 (11.5)	5 (14.7)	0.57 (0.18, 1.84)	0.285	0.72 (0.28, 1.88)	0.484	0.539
FHAD (−)	(*N* = 102)	(*N* = 79)	(*N* = 86)					
AD, *n* (%)	18 (17.6)	10 (12.7)	19 (22.1)	0.72 (0.35, 1.47)	0.387	1.25 (0.70, 2.23)	0.423	0.282
CMPA, *n* (%)	9 (8.8)	4 (5.1)	9 (10.5)	0.57 (0.18, 1.80)	0.336	1.19 (0.49, 2.85)	0.685	0.390
Model 3								
Lower % BM intake	(*N* = 0)	(*N* = 74)	(*N* = 101)					
AD, *n* (%)	N/A	9 (12.2)	25 (24.8)	N/A	N/A	N/A	N/A	0.032
CMPA, *n* (%)	N/A	7 (9.5)	13 (12.9)	N/A	N/A	N/A	N/A	0.499
Higher % BM intake	(*N* = 161)	(*N* = 31)	(*N* = 19)					
AD, *n* (%)	32 (19.9)	3 (9.7)	4 (21.1)	0.49 (0.16, 1.49)	0.106	1.06 (0.42, 2.67)	0.905	0.253
CMPA, *n* (%)	21 (13.0)	0 (0.0)	1 (5.3)	N/A	<0.001	0.40 (0.06, 2.83)	0.187	<0.001

AD: atopic dermatitis; CMPA: cow’s milk protein allergy, confirmed by oral food challenge; EBF: exclusive breastfeeding; pHF: partially hydrolyzed formula; SF: standard formula; PP: Per-Protocol; N: number of study participants; RR_1_: Relative risk for CMPA or AD EBF vs. pHF; RR_2_: Relative risk for CMPA or AD EBF vs. SF; CI: Confidence Interval; FHAD (+): family history of AD; FHAD (−): no family history of AD; Lower % BM intake: Percentage consumption of breast milk in the mixed-fed infants that is lower than or equal to the median average daily milk consumption (i.e., ≤41.8% of total milk consumption coming from breast milk or ≤278 mL of breast milk); Higher % BM intake: Percentage consumption of breast milk in the mixed-fed infants that is higher than the median average daily milk consumption (i.e., >41.8% of total milk consumption coming from breast milk or >278 mL of breast milk). Model 1 was adjusted for the potential confounding effect of gender, type of conception (i.e., normal vs. IVF), gestational age, type of delivery (i.e., labor vs. Cesarean), the amount of breast milk consumed by infants, the occurrence of early life infections, maternal and paternal educational level, maternal and paternal smoking at home, the presence of pets at home, the region of residence (i.e., urban vs. rural) and the country of infant’s birth. The rest of the models presented in the table were further adjusted for the interaction between treatment arm and FHAD (Model 2), and the amount of Breast Milk Intake by infants (Model 3). All *p*-values derived from Poisson Generalized Estimating Equation (GEE) regression analysis. *p*-value_1_ indicates the statistical significance of the treatment effect in the pHF compared to the EBF group; *p*-value_2_ indicates the statistical significance of the treatment effect in the SF compared to the EBF group; *p*-value_3_ indicates statistical significance of the overall treatment x time effect when comparing all treatment arms.

## Data Availability

The original contributions presented in the study are included in the article/Supplementary Material. Further inquiries can be directed to the corresponding author/s.

## Supplementary Materials

The following are available online at https://www.mdpi.com/article/10.3390/nu15112622/s1.
